# Supplementary material for: Seasonal niche tracking of climate emerges at the population level in a migratory bird
Source: Proc Biol Sci. 2020 Sep 23;287(1935):20201799. doi: 10.1098/rspb.2020.1799 (PMC7542805; doi:10.1098/rspb.2020.1799)
Supplement: SM1: Seasonal niche overlap; SM2: Seasonal niche tracking; SM3: Variability estimation for seasonal niche overlap and niche tracking [file rspb20201799supp1.docx]

# **Supplemental Material**

## **SM1. Seasonal niche overlap**

Table S1: Summary statistics of the seasonal niche overlap values (D) grouped by migratory flyway, by the individual or population-level analyses, weather or climate data, and by the environmental variable used (the three-dimensional space or single environmental variable).

| **level** | **environmental scale** | **overlap_type** | **migratory** | **n** | **mean** | **min** | **max** |
| --- | --- | --- | --- | --- | --- | --- | --- |
| individual | climate | multispace | east | 727 | 0.004 | 0.000 | 0.060 |
| individual | climate | multispace | west | 134 | 0.000 | 0.000 | 0.005 |
| individual | climate | NDVI | east | 709 | 0.175 | 0.000 | 0.714 |
| individual | climate | NDVI | west | 134 | 0.127 | 0.000 | 0.600 |
| individual | climate | precipitation | east | 727 | 0.127 | 0.000 | 0.695 |
| individual | climate | precipitation | west | 134 | 0.089 | 0.000 | 0.500 |
| individual | climate | temperature | east | 727 | 0.057 | 0.000 | 0.547 |
| individual | climate | temperature | west | 134 | 0.031 | 0.000 | 0.466 |
| individual | weather | multispace | east | 458 | 0.049 | 0.000 | 0.370 |
| individual | weather | multispace | west | 168 | 0.029 | 0.000 | 0.225 |
| individual | weather | NDVI | east | 458 | 0.269 | 0.000 | 0.789 |
| individual | weather | NDVI | west | 168 | 0.247 | 0.007 | 0.795 |
| individual | weather | precipitation | east | 320 | 0.604 | 0.078 | 0.911 |
| individual | weather | precipitation | west | 168 | 0.499 | 0.011 | 0.839 |
| individual | weather | temperature | east | 458 | 0.180 | 0.000 | 0.698 |
| individual | weather | temperature | west | 168 | 0.195 | 0.000 | 0.768 |
| population | climate | multispace | east | 72 | 0.037 | 0.000 | 0.146 |
| population | climate | multispace | west | 70 | 0.002 | 0.000 | 0.010 |
| population | climate | NDVI | east | 72 | 0.325 | 0.081 | 0.685 |
| population | climate | NDVI | west | 70 | 0.256 | 0.059 | 0.547 |
| population | climate | precipitation | east | 72 | 0.194 | 0.028 | 0.567 |
| population | climate | precipitation | west | 70 | 0.246 | 0.026 | 0.593 |
| population | climate | temperature | east | 72 | 0.118 | 0.024 | 0.531 |
| population | climate | temperature | west | 70 | 0.098 | 0.000 | 0.626 |
| population | weather | multispace | east | 72 | 0.190 | 0.002 | 0.369 |
| population | weather | multispace | west | 72 | 0.183 | 0.001 | 0.371 |
| population | weather | NDVI | east | 72 | 0.435 | 0.098 | 0.791 |
| population | weather | NDVI | west | 72 | 0.435 | 0.098 | 0.791 |
| population | weather | precipitation | east | 72 | 0.643 | 0.247 | 1.000 |
| population | weather | precipitation | west | 72 | 0.631 | 0.247 | 1.000 |
| population | weather | temperature | east | 72 | 0.358 | 0.085 | 0.730 |
| population | weather | temperature | west | 72 | 0.358 | 0.085 | 0.730 |

## **SM2. Seasonal niche tracking**

Figure S2: The proportion of significant niche tracking across seasons at individual/population level, with weather/climate variables and for each migratory route. Niche tracking was analysed using similarity tests (with n = 200 randomisations and a significance level of α=0.05). The colour of the line represents the scale of the environmental variable used (weather/climate), and the shape the migratory route (west/east). The niche tracking proportion was estimated in three-dimensional environmental space with the axes representing temperature, precipitation, and NDVI.


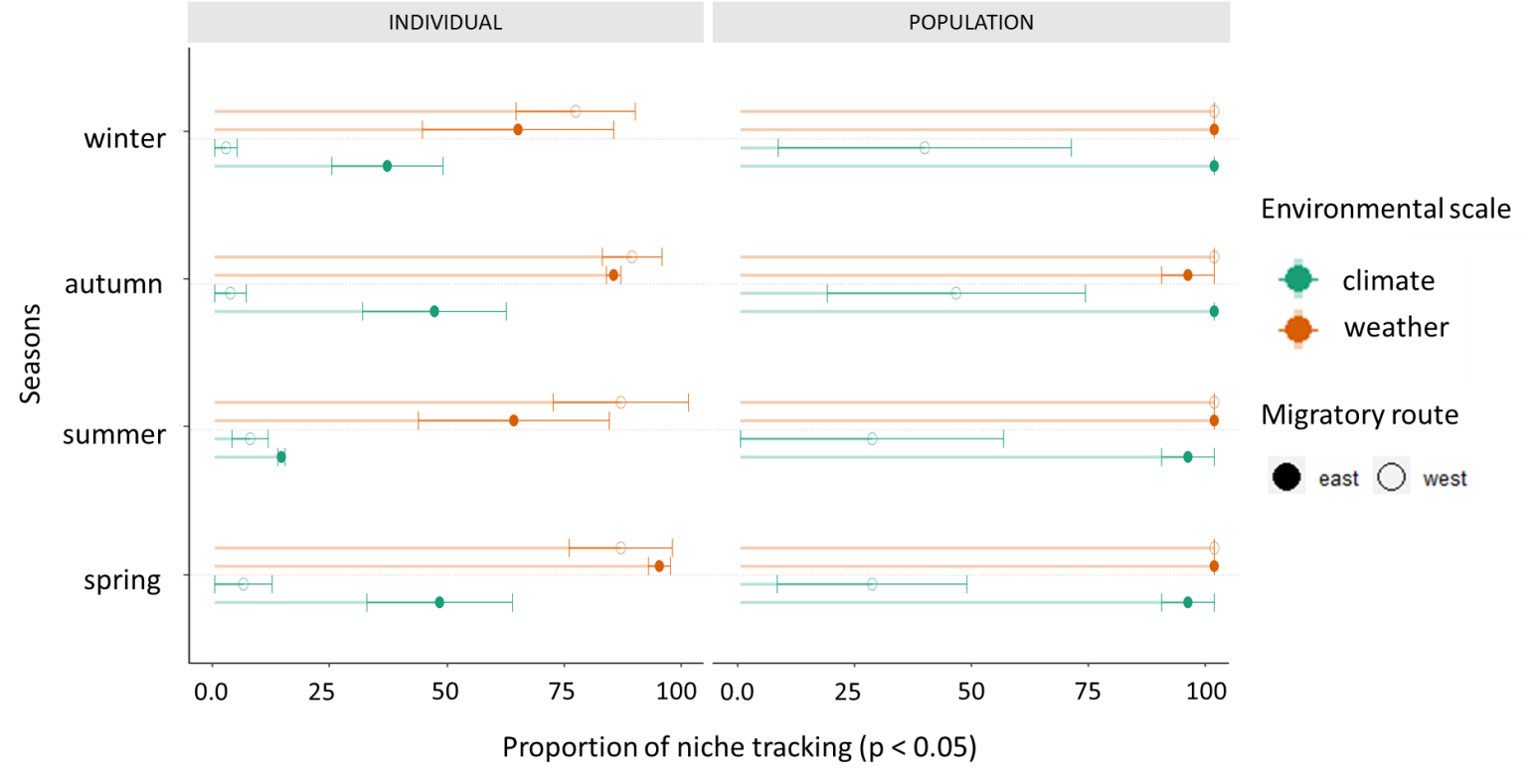


Table S2 Summary statistics of the proportion of cases that show significant niche tracking in the similarity analysis (p< 0.05), grouped by the individual or population level, weather or climate environmental data, and by the environmental variable used (three-dimensional space or each variable).

| **level** | **type** | **sim_type** | **Migratory route** | **n** | **median** | **mean** | **min** | **max** |
| --- | --- | --- | --- | --- | --- | --- | --- | --- |
| individual | climate | multispace | east | 12 | 31.06 | 36.11 | 12.82 | 73.42 |
| individual | climate | multispace | west | 12 | 0.00 | 4.82 | 0.00 | 18.18 |
| individual | climate | NDVI | east | 12 | 3.12 | 10.16 | 0.00 | 51.90 |
| individual | climate | NDVI | west | 12 | 11.81 | 10.96 | 0.00 | 27.27 |
| individual | climate | precipitation | east | 12 | 8.70 | 9.41 | 1.23 | 23.26 |
| individual | climate | precipitation | west | 12 | 0.00 | 7.18 | 0.00 | 33.33 |
| individual | climate | temperature | east | 12 | 5.10 | 7.43 | 1.28 | 17.95 |
| individual | climate | temperature | west | 12 | 0.00 | 3.40 | 0.00 | 12.50 |
| individual | weather | multispace | east | 12 | 84.21 | 76.31 | 23.08 | 97.37 |
| individual | weather | multispace | west | 12 | 89.29 | 83.93 | 57.14 | 100.00 |
| individual | weather | NDVI | east | 12 | 2.63 | 5.25 | 0.00 | 15.79 |
| individual | weather | NDVI | west | 12 | 0.00 | 2.38 | 0.00 | 7.14 |
| individual | weather | precipitation | east | 12 | 53.41 | 55.00 | 31.58 | 79.49 |
| individual | weather | precipitation | west | 12 | 64.29 | 67.86 | 42.86 | 100.00 |
| individual | weather | temperature | east | 12 | 7.89 | 9.19 | 0.00 | 23.68 |
| individual | weather | temperature | west | 12 | 7.14 | 6.55 | 0.00 | 21.43 |
| population | climate | multispace | east | 12 | 100.00 | 97.22 | 83.33 | 100.00 |
| population | climate | multispace | west | 12 | 16.67 | 35.00 | 0.00 | 100.00 |
| population | climate | NDVI | east | 12 | 0.00 | 0.00 | 0.00 | 0.00 |
| population | climate | NDVI | west | 12 | 0.00 | 0.00 | 0.00 | 0.00 |
| population | climate | precipitation | east | 12 | 0.00 | 6.94 | 0.00 | 50.00 |
| population | climate | precipitation | west | 12 | 0.00 | 0.00 | 0.00 | 0.00 |
| population | climate | temperature | east | 12 | 0.00 | 0.00 | 0.00 | 0.00 |
| population | climate | temperature | west | 12 | 0.00 | 4.17 | 0.00 | 16.67 |
| population | weather | multispace | east | 12 | 100.00 | 98.61 | 83.33 | 100.00 |
| population | weather | multispace | west | 12 | 100.00 | 100.00 | 100.00 | 100.00 |
| population | weather | NDVI | east | 12 | 0.00 | 0.00 | 0.00 | 0.00 |
| population | weather | NDVI | west | 12 | 0.00 | 0.00 | 0.00 | 0.00 |
| population | weather | precipitation | east | 12 | 50.00 | 58.33 | 33.33 | 83.33 |
| population | weather | precipitation | west | 12 | 50.00 | 58.33 | 33.33 | 83.33 |
| population | weather | temperature | east | 12 | 0.00 | 0.00 | 0.00 | 0.00 |
| population | weather | temperature | west | 12 | 0.00 | 0.00 | 0.00 | 0.00 |

## **SM3. Variability estimation for seasonal niche overlap and niche tracking**

Table S3: Posterior distribution estimates (mean and 95% confidence intervals) for the variability estimation result from the mixed models.

|  | **individual** | | | | **individual** | | | |
| --- | --- | --- | --- | --- | --- | --- | --- | --- |
|  | **weather** | | | | **climate** | | | |
|  | **niche overlap** | | **niche traking** | | **niche overlap** | | **niche traking** | |
|  | Post_mean | Conf. Interval | Post_mean | Conf. Interval | Post_mean | Conf. Interval | Post_mean | Conf. Interval |
| IIntercept | 0.109 | [-0.122, 0.359] | 95.331 | [38.438, 153.454] | 0.051 | [-0.063, 0.164] | -86.048 | [-147.239, -12.701] |
| migratory_divide(west) | -0.132 | [-0.458, 0.191] | 55.291 | [5.003, 113.875] | -0.33 | [-0.633, -0.039] | -181.757 | [-344.463, -19.049] |
| between_individuals | 0.2 | [0.021, 0.432] | 0.618 | [0.257 , 1.111] | 0.052 | [0, 0.112] | 0.618 | [0.257 , 1.111] |
| within_individuals | 0.858 | [0.764, 0.963] | 0.123 | [0.015 , 0.414] | 0.898 | [0.811, 0.989] | 0.123 | [0.015 , 0.414] |
| id_year | 0.04 | [0, 0.101] | 0.106 | [0 , 0.241] | 0.038 | [0, 0.089] | 0.106 | [0 , 0.241] |
| dic | 1708.054 | | 19.427 | | 2388.779 | | 24.249 | |
| repeatibility | 0.108 | [0.025, 0.239] | 0.068 | [0.029, 0.244] | 0.03 | [0.014, 0.110] | 0 | [0, 0.266] |
| n | 626 | | 626 | | 861 | | 861 | |
|  | **population** | | | | **population** | | | |
|  | **weather** | | | | **climate** | | | |
|  | **niche overlap** | | **niche traking** | | **niche overlap** | | **niche traking** | |
| IIntercept | -0.001 | [-10.520, 12.482] | 201.777 | [-242.117, 757.440] | 3.194 | [-18.231, 22.223] | 136.786 | [-1498.906, 1258.909] |
| between_populations | 0.853 | [0.00, 0.057] | 3848340.00 | [0.0001 , 769401.200] | 0.853 | [0.00, 0.057] | 9,537,697.00 | [14.641, 6265044.000] |
| within_populations | 0.393 | [0.326,0.460 ] | 3844.83 | [0.061, 14924.820 ] | 0.393 | [0.326,0.460 ] | 11,499.21 | [11.204, 28493.070 ] |
| pop_year | 0.069 | [0.010,0.161 ] | 3926.87 | [0.00,18423.090 ] | 0.069 | [0.010,0.161 ] | 373.295 | [0.00002, 1728.018 ] |
| dic | 384.46 | | 2.878 | | 555.265 | | 6.271 | |
| repeatibility | 0.994 | [0.003, 1] | 0.999 | [0.962, 1] | 0.997 | [0.299, 1] | 0.999 | [0.962, 1] |
| n | 144 | | 144 | | 142 | | 142 | |
